# The link between obesity and vitamin D in bariatric patients with omega-loop gastric bypass surgery - a vitamin D supplementation trial to compare the efficacy of postoperative cholecalciferol loading (LOAD): study protocol for a randomized controlled trial

**DOI:** 10.1186/s13063-015-0877-9

**Published:** 2015-08-05

**Authors:** Maria Luger, Renate Kruschitz, Rodrig Marculescu, Helmuth Haslacher, Friedrich Hoppichler, Enikö Kallay, Christian Kienbacher, Carmen Klammer, Melanie Kral, Felix Langer, Eva Luger, Gerhard Prager, Michael Trauner, Stefan Traussnigg, Tanja Würger, Karin Schindler, Bernhard Ludvik

**Affiliations:** Division of Endocrinology and Metabolism, Department of Internal Medicine III, Medical University of Vienna, Währinger Gürtel 18-20, 1090 Vienna, Austria; Special Institute for Preventive Cardiology and Nutrition – SIPCAN, Guggenbichlerstraße 8/15, 5026 Salzburg, Austria; Clinical Institute for Medical and Chemical Laboratory Diagnostics, Department of Laboratory Medicine, Medical University of Vienna, Währinger Gürtel 18-20, 1090 Vienna, Austria; Department of Pathophysiology and Allergy Research, Medical University of Vienna, Währinger Gürtel 18-20, 1090 Vienna, Austria; Division of Gastroenterology and Hepatology, Department of Internal Medicine III, Medical University of Vienna, Währinger Gürtel 18-20, 1090 Vienna, Austria; Division of General Surgery, Department of Surgery, Medical University of Vienna, Währinger Gürtel 18-20, 1090 Vienna, Austria; Institute of Social Medicine, Centre for Public Health, Medical University of Vienna, Kinderspitalgasse 15/1, 1090 Vienna, Austria; Department of Pathology, Medical University of Vienna, Währinger Gürtel 18-20, 1090 Vienna, Austria

**Keywords:** Bariatric patients, Cholecalciferol, Obesity, Vitamin D, Vitamin D supplementation trial

## Abstract

**Background:**

Beyond its classical role in calcium homoeostasis and bone metabolism, vitamin D deficiency has been found to be associated with several diseases, including diabetes, non-alcoholic fatty liver disease, and even obesity itself. Importantly, there are limited data on therapeutic strategies for vitamin D deficiency in bariatric patients, and the procedure-specific guidelines may not be sufficient. To improve long-term outcomes, nutritional screening and appropriate supplementation to prevent nutrient deficiencies are urgently needed. Therefore, the aim of this study is to examine effects and safety of a forced dosing regimen of vitamin D versus conventional dose supplementation on vitamin D levels and other parameters in bariatric patients.

**Methods/Design:**

The study includes loading plus repeat dosing compared with repeated administration of vitamin D without a loading dose, according to guidelines, in a prospective, double-blind, randomized controlled trial. Up to a triple oral loading dose is given on day 1, then 2 and 4 weeks after surgery (100,000 IU dose each time), followed by an oral maintenance dose (3420 IU/day). The control group (*n* = 25) will receive placebo, followed by administration of a standard dose (3420 IU/day). We hypothesize that a significant increase in vitamin D levels will occur in patients in the treatment group (*n* = 25) by 24 weeks after surgery. Further measurements are aimed at evaluating changes in inflammation, bone turnover, insulin resistance, blood pressure, liver, mental health, and gut microbiota of patients undergoing omega-loop gastric bypass surgery. Furthermore, possible associations between concentrations of vitamin D, the involved enzymes, or vitamin D receptor in adipose and/or liver tissues will be determined.

**Discussion:**

To our knowledge, this trial is the first of its kind with this type of vitamin D supplementation in bariatric patients. Its major strength is the design and implementation of evaluation of influencing factors such as liver function, bone health, inflammation, insulin resistance, blood pressure, symptoms of depression, or microbiota. This alternative vitamin D dosing regimen has the potential to be a safe, fast, evidence-based treatment of vitamin D deficiency in bariatric patients. Owing to the increasing number of bariatric patients, it is also of interest to elucidate the link between obesity and vitamin D.

**Trial registration:**

ClinicalTrials.gov identifier: NCT02092376. Registered on 17 March 2014.

## Background

Obesity is a global epidemic [1] that is associated with various comorbidities. It is part of the definition of the metabolic syndrome, including arterial hypertension and disturbances in glucose and lipid metabolism, with an inherent risk of development of cardiovascular disease [2, 3]. These associated issues and obesity itself contribute to increased morbidity, mortality rates, and health care costs [4, 5].

Weight reduction is not easy to achieve and maintain. Consequently, many individuals, especially those with comorbidities, are referred for bariatric weight loss surgery as a way to treat obesity and (pre)diabetes successfully [6]. Bariatric surgery is associated with effective and long-term weight loss in morbidly obese patients and decreases overall mortality [7]. At present, there are three categories of bariatric procedures: (1) purely gastric restriction (e.g., gastric banding); (2) gastric restriction with some malabsorption, as represented by the Roux-en-Y (RYGB) or omega-loop gastric bypass (OLGB), reducing the intake and absorption of food [8]; and (3) gastric restriction with significant intestinal malabsorption (e.g., biliopancreatic diversion) [9]. In general, the more complex the procedure, the better are the results in terms of weight loss [10]. Because of this situation and the fact that obesity itself is often accompanied by nutritional deficiencies, bariatric patients often have perioperative nutrient deficiencies [9, 11–18]. These deficiencies should be detected and clinically addressed early to avoid postoperative complications. To improve long-term outcomes following bariatric surgery, nutritional screening and prescribing appropriate supplementation to prevent nutrient deficiencies is recommended at an early stage in pre- and postoperative care owing to the malabsorption and insufficient intake. Vitamin D deficiency is common following bariatric surgery and has been reported to occur in 50–80 % of bariatric patients [19–22]. There are limited data on how best to treat low vitamin D status in bariatric patients. Procedure-specific guidelines may not be helpful in daily practice, and the updated guidelines have not been evaluated yet [23].

Recent studies have shown that vitamin D may have an impact on the prevention of many diseases, including autoimmune disorders, hypertension, cancers, diabetes [3, 24–26], depression [27–29], and, even more theoretically, probably obesity itself [30].

Obesity is frequently characterized by reduced vitamin D bioavailability, insulin resistance, and a chronic inflammatory response. There is a relationship between serum concentrations of 25-hydroxy-vitamin D (25-OHD) and several circulating inflammatory markers in severely obese individuals [31]. This observed relationship between low vitamin D status and obesity may be caused by mechanisms such as sequestration in adipose tissue. The mechanisms underlying the inverse relationship between obesity and vitamin D deficiency are largely unknown. Low serum 25-OHD might contribute to obesity by affecting lipogenesis and/or adipogenesis in the adipose tissue. Interestingly, recent studies [32] suggest that the adipose tissue could be a direct target of vitamin D and that the hormone may modulate adipose tissue formation and function [33–37].

Obesity has recently been associated with non-alcoholic fatty liver disease (NAFLD). NAFLD comprises a disease spectrum ranging from relatively benign hepatic steatosis to more severe steatohepatitis, fibrosis, cirrhosis, and ultimately liver cancer [38]. It has become the most common form of chronic liver disease in Western countries, with a prevalence approaching 50 % of the population (already 40 % in the European Union) [39, 40]. It is estimated that NAFLD will be the number 1 indication for liver transplants in the United States in 2020 [41]. Currently, surgical treatment of coexisting NAFLD in morbidly obese patients is an evolving matter of debate [42]. A few studies propose that low levels of vitamin D may contribute to the development of NAFLD [43]. However, the mechanisms underlying the association of vitamin D and NAFLD are not yet fully understood. Recent animal studies have shown that vitamin D has an important role in the regulation of oxidative stress, the production of proinflammatory cytokines [44, 45], hepatocyte apoptosis [46], and even liver fibrosis [47]. Furthermore, NAFLD is present in approximately 90 % of bariatric patients [48, 49], and up to 5 % of these patients may have unsuspected cirrhosis [50–52]. Because NAFLD is associated with the presence of obesity, glucose and/or lipid disturbances, and arterial hypertension, it is now considered the hepatic manifestation of the metabolic syndrome.

There are limited data on therapeutic strategies for vitamin D deficiency in bariatric patients, and procedure-specific guidelines may not be very helpful in daily clinical practice. Nevertheless, in the 2013 clinical practice guidelines [23], the American Association of Clinical Endocrinologists, the Obesity Society and American Society for Metabolic and Bariatric Surgery recommend a vitamin D supplementation of at least 3000 IU per day and to titrate to the therapeutic 25-OHD level of >75 nmol/L. This recommendation outlines the best evidence level (grade A) [23], which is based on a single randomized controlled trial done in the United States [53]. In that study, the authors attempted to find the optimal vitamin D treatment dose after surgery. Patients who had undergone RYGB were randomized to three doses of cholecalciferol: 800 IU, 2000 IU, and 5000 IU per day. The authors found that all groups had insufficient vitamin D levels at baseline and after 12 months; sufficient vitamin D concentrations were reached in only 44, 78, and 70 % of the patients in the respective groups [53]. Thus, even with very high-dose supplementation, it may be challenging to achieve normal 25-OHD levels in bariatric patients [54].

Vitamin D has multiple physiological functions beyond its classical role in calcium homoeostasis and bone metabolism, and morbidly obese persons have an increased risk for low circulating 25-OHD levels [55, 56] owing to the storage of vitamin D in adipose tissue [55].

Therefore, randomized controlled trials with vitamin D supplementation, such as the present study (Link between Obesity And Vitamin D in bariatric patients with omega-loop bypass surgery [LOAD]), are needed to establish and further pursue evidence-based and potentially optimal treatment regimens for the increasing number patients with low vitamin D status following bariatric surgery and to define more carefully the impact of specific surgical factors on vitamin D status.

## Methods/Design

### Overview

**Fig. 1:**
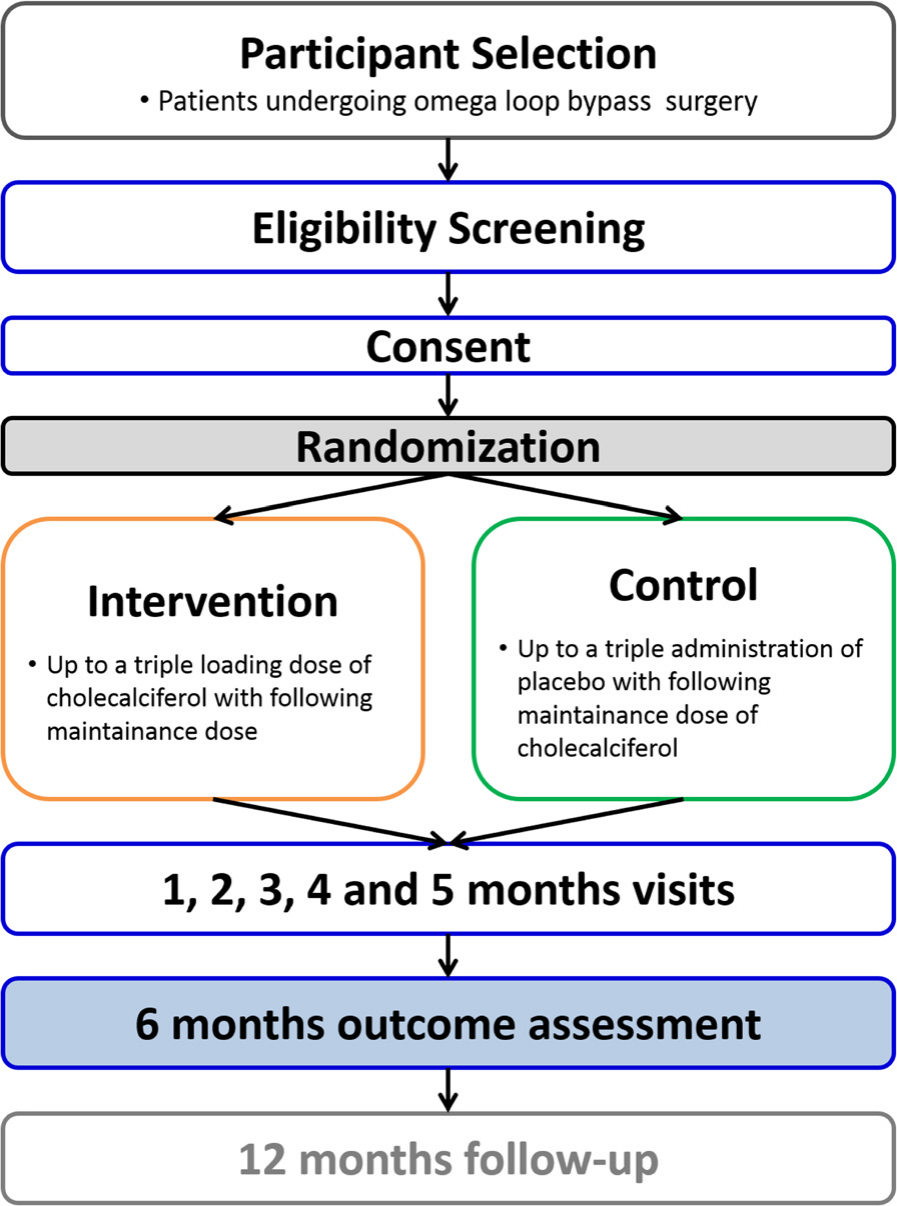
Study design and assessment points

This prospective, double-blind, randomized controlled intervention trial includes administration of a vitamin D loading dose and repeat doses in patients undergoing bariatric surgery. A stratified randomization design is being used to obtain comparable groups to ensure that baseline variables (25-OHD, age, and sex) are evenly distributed between groups. Subjects are randomly assigned to the intervention or control group. In this study, 50 bariatric patients planning to undergo OLGB will be recruited. Over the first month postoperatively (day 1–3, weeks 2 and 4), they will receive the loading dose of vitamin D. Afterwards, the maintenance dose will be given up to 24 weeks and until the follow-up visit. An overview of the study design and the assessment points is provided in Fig. 1. The study was approved by the local ethics committee of the Medical University of Vienna (reference number 1899/2013) by the Austrian Competent Authority (reference number LCM-718280-0001), and it complies with the Declaration of Helsinki [57]. Furthermore, the protocol was registered at ClinicalTrials.gov (identifier: NCT02092376) and EudraCT (identifier: 2013-003546-16). The study methods are in accordance with the 2010 Consolidated Standards of Reporting Trials (CONSORT) Statement guidelines for reporting randomized trials [58].

### Preevaluation

The methods in this study are built upon a cohort study in which investigators evaluated inter alia the vitamin D status of 50 morbidly obese patients who underwent OLGB [54]. The findings of the evaluation illustrated that 96 % had vitamin D deficiency preoperatively and that, after non-standardized vitamin D supplementation, 80 % still had vitamin D deficiency 12 months postoperatively [54]. Additionally, there is evidence that, despite forced vitamin D supplementation, some patients are still at risk for increased bone resorption [22, 59, 60]. It could be possible that morbidly obese patients might need higher 25-OHD levels to reduce the risk for developing metabolic bone disease, osteoporosis [61], or other associated disorders.

### Study objectives

#### Primary outcome measures

The primary objective of the present study is to examine whether administration of up to three oral loading doses in the first month postoperatively (day 1 and at weeks 2 and 4), followed by an oral maintenance dose (intervention group) in bariatric patients can significantly increase 25-OHD levels 24 weeks after surgery as compared with a control group receiving placebo followed by the standard daily maintenance dose (control group).

#### Secondary outcome measures

In further measurements, changes in inflammation, bone turnover, bone mineral density (BMD), insulin resistance, blood pressure, body fat mass, stage of liver stiffness and steatosis, and mental health (improvement of depression) will be investigated. Furthermore, we will assess the adipose depot vitamin D concentrations and expression of enzymes (25-hydroxylases such as CYP27A1, CYP2R1, CYP2J2, CYP3A4, CYP2C11, and CYP27B1, as well as 24-hydroxylase CYP24A1) and vitamin D receptor in subcutaneous tissue (SAT), visceral adipose tissue (VAT), and liver tissue samples collected during surgery. Additionally, the liver tissue samples will be used in addition to the prescribed study-related measures for a histological examination as extended diagnostics for NAFLD or non-alcoholic steatohepatitis and molecular analysis to further evaluate disease specific mechanisms [62, 63].

### Recruitment and eligibility

We are recruiting bariatric patients planning to undergo OLGB and who are in- and/or outpatients in the obesity clinic at the Department of Internal Medicine III or the Department of Surgery at Vienna General Hospital. The inclusion and exclusion criteria are shown in Table 1.

**Table 1:** Inclusion and exclusion criteria

Inclusion	Exclusion
Men and women ages 18–100 years	Another planned form of bariatric surgery
Planned OLGB	Hypercalcemia (calcium >2.63 mmol/L) or hypocalcemia (calcium <1.75 mmol/L)
25-OHD <75 nmol/L	Renal insufficiency (creatinine >133 μmol/L or GFR <50 ml/min)
BMI >40 or ≥35 kg/m^2^ with comorbidities	Primary hyperparathyroidism
Body weight <150 kg (owing to limitation of DEXA measurement)	Malignancy
Capability to consent	Infection (e.g., HIV)
	Medical conditions requiring daily calcium supplements or antacid use
	Known hypersensitivity to cholecalciferol
	No capability to consent
	Imprisoned persons

*Abbreviations*: *BMI* body mass index, *DEXA* dual-energy X-ray absorptiometry, *GFR* glomerular filtration rate, *25*-*OHD* 25-hydroxy-vitamin D, *OLGB* omega-loop gastric bypass

**Table 2:** Measurements at several time points

	Name	Screening		Intervention								Follow-up
	Duration	12 months		6 months								6 months
Visits	Time		Surgery	Days 1–3	Wk 2	Wk 4	Wk 8	Wk 12	Wk 16	Wk 20	Wk 24	Wk 48
Anamnesis	Anamnesis	X									X	X
	Body weight	X			X	X	X	X	X	X	X	X
	Anthropometry	X				X	X	X	X	X	X	X
	Dietary assessment, BDI	X				X		X			X	X
Bone density	DEXA	X									X	X
Liver and microbiota	FibroScan and CAP^TM^	X				X		X			X	X
	Stool samples	X				X		X			X	X
Biopsies	Liver, SAT, VAT		X									
Supplementation	Loading dose			X	X	X						
	Maintenance dose					X	X	X	X	X	X	X
Blood parameter	Vitamin D	X			X	X	X	X	X	X	X	X
	Biochemical	X			X	X	X	X	X	X	X	X
	Liver	X				X		X			X	X
	Inflammation	X			X	X	X	X	X	X	X	X
	Insulin resistance	X			X	X	X	X	X	X	X	X
	Bone turnover	X				X		X			X	X

*Abbreviations*: *BDI* Beck Depression Inventory, *CAP* controlled attenuation parameter; *DEXA* dual-energy X-ray absorptiometry, *SAT* subcutaneous adipose tissue, *VAT* visceral adipose tissue

### Sample size calculation

The study is powered to detect differences among the groups for 24-week serum 25-OHD levels. On basis of the preevaluation (cohort study), the placebo group was conservatively estimated to have an average 24-week 25-OHD level of 50 ± 30 nmol/L. For sample size calculation, the difference in 25-OHD levels between groups will be considered. Given a clinically relevant difference of 30 nmol/L and a standard deviation of 35 nmol/L of the differences, a two-sided significance level of 0.05, a sample size of 22 per group is needed to reach 80 % statistical power. Because imputation for dropouts of 20 % may have some inestimable effect on the assumed standard deviation of the differences, the sample size is increased to 25 per group. To reach this sample size, a total of 50 bariatric patients in the study are needed. The primary endpoint will be analyzed according to the intention-to-treat principle.

### Randomization

Subjects are randomly assigned to the intervention or control group, stratified by 25-OHD, age, and sex using the Randomizer for Clinical Trials 1.8.1 online tool [64]. Each subject receives an appropriate randomization number. Randomization is carried out after the patient has signed the informed consent form and before undergoing bariatric surgery, right before the baseline assessment. Allocation to the groups is performed with consecutively numbered dark bottles containing either vitamin D_3_ (cholecalciferol) or placebo (carrier oil). These allocation bottles are labeled with the randomization number.

### Intervention

#### Loading dose determination

A loading dose is an extent of drug or substance designed to fill the central volume of distribution to a concentration that matches the final plateau concentration achieved with the maintenance dose. Consequently, this final plateau could be achieved sooner than the four half-lives required if it is administered only at the maintenance dose rate [65]. According to the current guidelines, a minimal daily vitamin D supplementation of at least 3000 IU is recommended [23]. To prevent failure of the study due to an insufficient treatment dose, we chose the maximum loading dose for which sufficient safety data are available. The functional half-life for vitamin D_3_ in the body is between 2 and 3 months [66–68]. As a result, the loading dose can be calculated as the cumulative maintenance dose that is planned to be given through one functional half-life of vitamin D, as follows:

$$ \mathrm{Loading}\ \mathrm{dose}=\left(\mathrm{daily}\ \mathrm{maintenance}\ \mathrm{dose}\ \mathrm{of}\ 3420\ \mathrm{IU}\right)\times \left(90\ \mathrm{days}\right)=307,800\ \mathrm{IU}\left(\mathrm{total}\ \mathrm{loading}\ \mathrm{dose}\right) $$

In previous studies, researchers used doses exceeding this dosage, at 500,000 and 600,000 IU of cholecalciferol/vitamin D_3_ in frail elderly patients, young patients, and critically ill patients, without observing any adverse effects (e.g., hypercalcemia) [66, 69, 70]. The total loading dose of 300,000 IU is given on the basis of the measured 25-OHD blood levels and/or concentrations (except for the first dose, given at day 1 after surgery, of 100,000 IU) up to a maximum of 300,000 IU. To make the loading dose protocol safer, 100,000 IU of vitamin D is administered at one time to allow clearance from the circulation between each increment of the loading dose [71].

According to the European Food Safety Authority (EFSA), the no observed adverse effect level was established at 10,000 IU per day and the tolerable upper intake level (UL) at 4000 IU per day (based on induction of hypercalcemia as the indicator of toxicity) [72, 73]. The maintenance dose, given after the loading dose, is 3420 IU per day, which is in accordance with the EFSA panel guideline of a UL of 4000 IU per day [72]. Taken together, no harm or risk is expected for patients in the intervention and placebo groups. Importantly, as a result, appropriate selection of maintenance and loading doses can reduce the need for follow-up monitoring of 25-OHD levels.

#### Intervention group

**Fig. 2:**
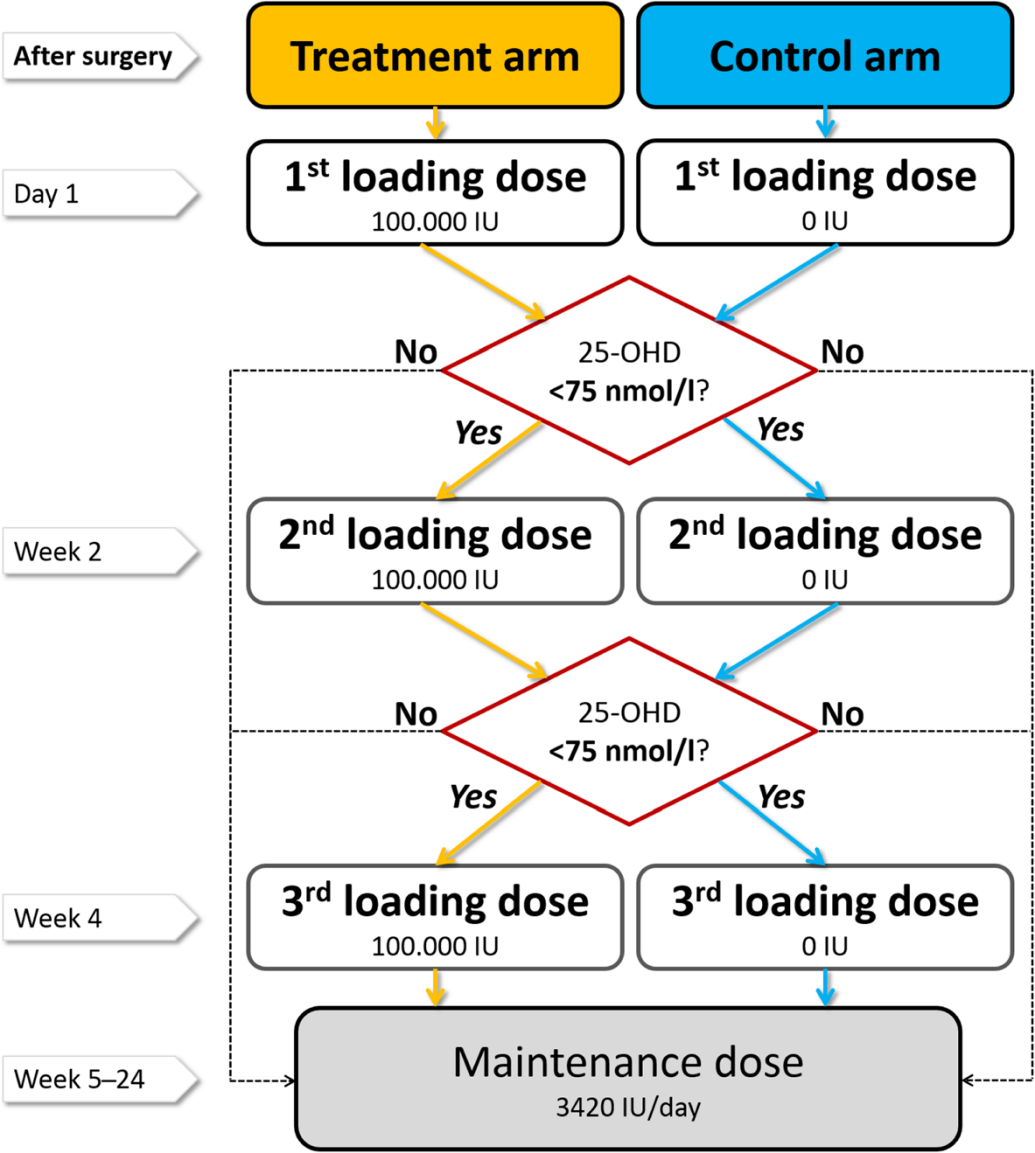
Dosing regimen. [25 - OHD 25-hydroxy vitamin D]

The oral loading dose of 300,000 IU cholecalciferol (vitamin D_3_) is divided into three doses (100,000 IU each) and is given on day 1 and at 2 and 4 weeks postoperatively. The oral maintenance dose is given up to 24 weeks and until the follow-up visit (46 weeks). All patients in the intervention group receive the first loading dose. The second and third doses are given based on the 25-OHD serum concentration, which is assessed before administration. If the 25-OHD does not reach a level above 75 nmol/L, a 100,000 IU dose is again given, up to 300,000 IU in total. After the last loading dose, a maintenance dose of 3420 IU per day (approximately translating to 60 drops and 24,000 IU per week) should maintain the high 25-OHD concentration, according to guidelines (Fig. 2) [23].

#### Placebo group

The carrier solution (medium-chain triglyceride oil [MCT]), of the verum formulation (MCT + vitamin D) is used in the placebo-only group. The placebo group is treated exactly the same as the intervention group (Fig. 2).

### Measurements

**Fig. 3:**
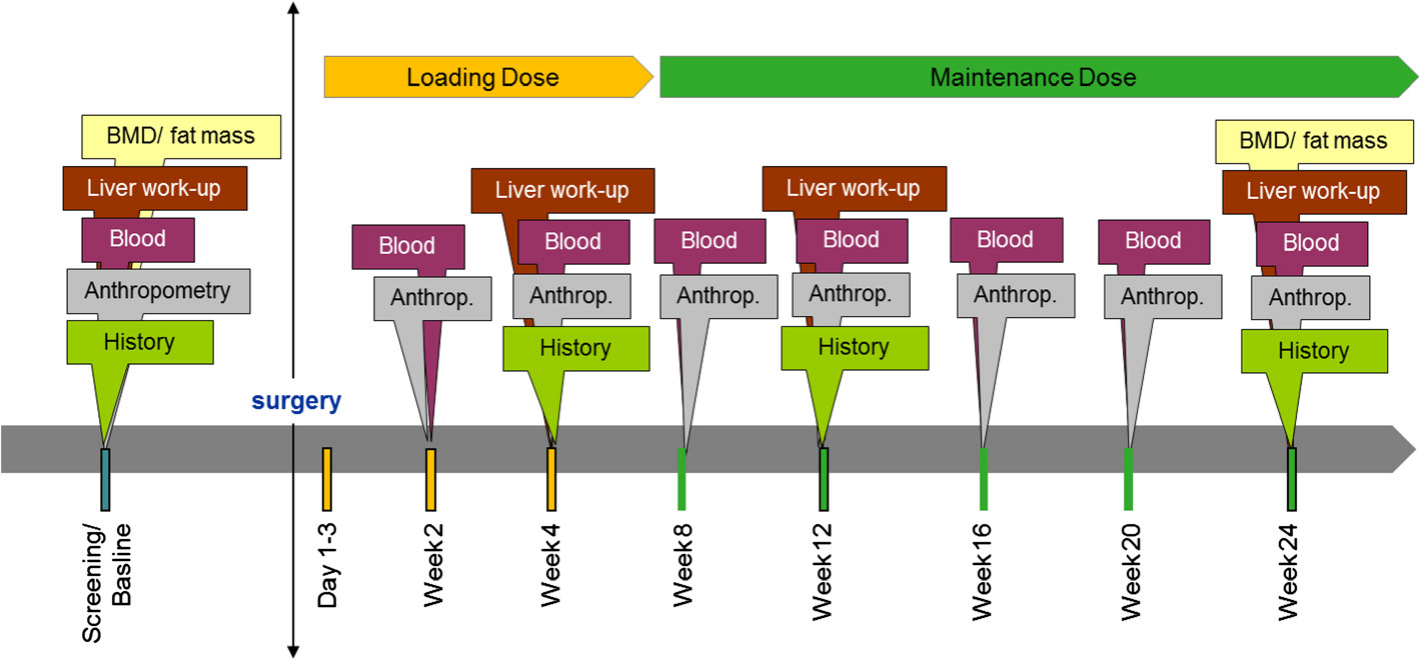
Flowchart. [BMD bone mineral density]

Patient history and dietary assessment data are documented at baseline and collected four times. For all subjects, body weight, body composition, and blood parameters are assessed eight times. The liver function assessment is done four times. Blood pressure, depression symptoms, BMD, and total body fat mass are measured two times in all study groups. The visit and assessment schedule are shown in Fig. 3 and Table 1.

The measurements to be performed are described in the subsections below and in Table 2.

#### History, blood pressure, and dietary assessment

1. Medical history, such as comorbidities, prescribed medications, and demographics (marital status, education, migration background, and career)
2. Blood pressure (mmHg)
3. Dietary assessment to provide information regarding dietary vitamin D intake as follows:
  - *5*-*day food record*: During the week before the scheduled visit, patients should record their food intake for 5 days. Data are analyzed using the nutritional software nut.s, which is based on Bundeslebensmittelschlüssel (Max Rubner-Institut; http://www.bls.nvs2.de/).
  - *Mediterranean Adherence Score*: A brief, validated questionnaire regarding adherence to the Mediterranean diet results on a 14-point scale [74]; the higher the score, the better the adherence to the proposed diet

#### Anthropometry

1. Weight (kg), measured with a calibrated scale
2. Height (cm), measured with a wall stadiometer
3. Waist circumference, measured with an inelastic tape
4. *Body composition*: fat-free mass, lean body mass, total body water, and phase angle are assessed by bioelectrical impedance analysis (BIA) [75], based on the transfer of a low-voltage alternating current through the body. This measurement captures the voltage drop of the applied current and result in measuring resistance and reactance. It is used with height, weight, age, and sex in a number of multiple regression relationships to calculate body composition compartments [76]. A single frequency BIA at 50 kHz and 0.8 mA (Biacorpus RX 4000; MediCal HealthCare GmbH, Karlsruhe, Germany) is passed between electrodes (Bodystat, Isle of Man, UK) placed on the dominant side hand and foot with patients in the supine position.

#### Stage of liver stiffness and steatosis

Transient elastography FibroScan (Echosens, Paris, France) with a controlled attenuation parameter (CAP™) will be performed based on transmitting a vibration signal into the liver and receiving the resulting shear wave, which depends on liver stiffness. CAP™ measures ultrasonic attenuation in the liver at 3.5 MHz and detects liver steatosis.

#### Bone mineral density and total body fat mass

Dual-energy X-ray absorptiometry (DEXA) will be used to measure bone mineral content, BMD, and percent body fat.

#### Laboratory parameters

1. *Vitamin D status*: 25-OHD (nmol/L), 1,25-dihydroxy vitamin D (pg/ml)
2. *Biochemical parameters*: sodium (nmol/L), potassium (nmol/L), chloride (nmol/L), calcium (nmol/L), magnesium (nmol/L), iron (μg/dl), total protein (g/L), albumin (g/L), triglyceride (mg/dl), total cholesterol (mg/dl), high-density lipoprotein (mg/dl), low-density lipoprotein (mg/dl), vitamin B_12_ (pmol/L), folic acid (nmol/L), vitamin A (μmol/L), vitamin E (μmol/L), ferritin (μg/L), transferrin (mg/dl), and transferrin saturation (%)
3. *Bone turnover markers*: calcium (mmol/L), osteocalcin (ng/ml), bone-specific alkaline phosphatase (U/L), type I collagen cross-linked C-terminal telopeptide (ng/ml), anorganic phosphate (mmol/L), parathyroid hormone intact (pg/ml), and amino-terminal propeptide of type I procollagen (ng/ml)
4. *Inflammation markers*: C-reactive protein (mg/dl) and interleukin 6 (pg/ml)
5. *Insulin resistance markers*: C-peptide (ng/ml), insulin (μU/ml), blood glucose (mg/dl), and glycosylated hemoglobin HbA1c (%)
6. *Liver parameters*: aspartate transaminase (U/L), alanine transaminase (U/L), γ-glutamyl transpeptidase (U/L), total alkaline phosphatase (U/L), and cholinesterase (U/L)
7. Blood count
8. *Coagulation*: Normotest (%) (Technoclone, Vienna, Austria), activated partial thromboplastin time (s), and fibrinogen (mg/dl)
9. *Protein electrophoresis*: serum protein electrophoresis, haptoglobin (mg/dl), and α_2_-macroglobulin (mg/dl)

#### Depression symptoms

The Beck Depression Inventory (BDI) [77] (simplified BDI-V), a 21-question, multiple-choice, self-report inventory will be used for measuring the severity of depression [78].

#### Microbiota composition

Stool samples will be collected to evaluate changes in the composition of intestinal microbiota resulting from weight reduction and vitamin D supplementation.

#### Biopsy

For the purposes of describing adipose depot vitamin D concentrations and expression of enzymes in SAT, visceral adipose VAT and liver tissue samples are collected by the surgeon during surgery. Furthermore, liver tissue samples are used, in addition to the prescribed study-related measures, for histological examination as extended diagnostics. Samples are snap-frozen in isopentane and stored in nitrogen tanks at −196 °C until assayed. Biopsies will be performed as follows:

1. *Adipose tissue*: The SAT sample is extracted through a linear incision in the abdominal wall with no additional incision. The VAT sample is taken from the greater omentum, coming off the transverse colon.
2. *Liver tissue*: Standard intraoperative wedge and needle biopsies are collected during surgery.

### Statistical analysis

Data exploration using descriptive statistical analysis and inferential statistics (uni- and multivariate) will be performed. The data will be described by frequencies or percentages, means and standard deviations, medians and confidence intervals, and graphics. For proofing of normal distributions, visual inspection of histograms or box plots will be used. Independent samples *t* tests, Mann–Whitney *U* test or *χ*^2^ tests will be performed to compare groups. Correlation coefficients will be used with respect to the secondary outcomes. Analysis of covariance will be used to compare parameters at baseline and after the intervention (postoperatively), as well as after the follow-up, between intervention and control groups, adjusting for the baseline value as a covariate. IBM SPSS Statistics for Windows version 22 software (IBM, Armonk, NY, USA) will be used for all statistical analyses. All tests are two-sided, and a *p* value <0.05 is considered statistically significant.

## Discussion

The major strengths of the proposed study are the prospective, randomized, double-blind, placebo-controlled design and the implementation of an independent evaluation of outcome parameters. The expected result, positive or negative, should provide an evidence-based and potentially optimal treatment of vitamin D deficiency following bariatric surgery and should define the interactions of vitamin D and specific factors, such as liver and bone health, inflammation, insulin resistance, blood pressure, depression symptoms, microbiota, and weight loss. Furthermore, as far as we know, the LOAD study is one of the first trials with this type of vitamin D supplementation in bariatric patients.

Some aspects of the study design, however, may deserve a closer discussion. Why was an active treatment regime after the loading dose chosen for the control group? From an ethical point of view, and bearing in mind the importance of vitamin D for bone metabolism, it would be difficult not to provide these patients with vitamin D supplementation at all. Therefore, we decided to use the placebo only for the loading dose (in the first postoperative month) to compare this dosing regimen with the usual supplementation. Indeed, with this study design, we expect to treat and provide supplements to the patients more effectively than in routine care.

The prospective benefit will have a positive impact on vitamin D status and therefore on the well-being and health-related quality of life, which has been described in the literature [79–81]. A further question might be why a supplementation regimen different from an established treatment of vitamin D deficiency in bariatric patients was chosen. In fact, from a methodological point of view, a constant daily supplementation of vitamin D would be preferable. However, there is evidence in the literature that this standard supplementation in bariatric patients is not sufficient, and with this novel dosing regimen, the final vitamin D plateau could be achieved earlier than the four half-lives required when administered only at the maintenance dose rate [65]. Another important point for the success of the LOAD study is the compliance of the participants regarding regular study visits. Hence, we are in close contact with the participants, and they are receiving text messages before the scheduled appointment.

Finally, one might assume that the rather small sample size of 50 bariatric patients in the proposed study might be underpowered to detect a significant difference between groups. However, the study is powered to detect differences between the groups for 24-week serum 25-OHD levels, with a given clinically relevant difference of 30 nmol/L and a standard deviation of 35 nmol/L of the differences. Thus, if high-dose supplementation with vitamin D is indeed effective, a reduction of vitamin D deficiency after 24 weeks would not be unrealistic.

In conclusion, this vitamin D supplementation regimen has the potential to be a safe, orally available, cheap, and evidence-based treatment of low vitamin D status following bariatric surgery in this increasingly prevalent group, and the study probably also is an opportunity to find an association between obesity and vitamin D deficiency.

## Trial status

The LOAD study commenced recruitment in April 2014. Recruitment was completed in April 2015.

## Abbreviations

BDI: Beck Depression Inventory
BIA: Bioelectrical impedance analysis
BMD: Bone mineral density
BMI: Body mass index
CAP: Controlled attenuation parameter
DEXA: Dual-energy X-ray absorptiometry
EFSA: European Food Safety Authority
GFR: Glomerular filtration rate
LOAD: Link between Obesity And Vitamin D in bariatric patients with omega-loop bypass surgery
MCT: Medium-chain triglyceride
NAFLD: Non-alcoholic fatty liver disease
25-OHD: 25-hydroxy vitamin D
OLGB: Omega-loop gastric bypass
RYGB: Roux-en-Y gastric bypass
SAT: Subcutaneous adipose tissue
UL: Tolerable upper intake level
VAT: Visceral adipose tissue
